# Nanopore Sequencing in Blood Diseases: A Wide Range of Opportunities

**DOI:** 10.3389/fgene.2020.00076

**Published:** 2020-02-19

**Authors:** Crescenzio Francesco Minervini, Cosimo Cumbo, Paola Orsini, Luisa Anelli, Antonella Zagaria, Giorgina Specchia, Francesco Albano

**Affiliations:** Department of Emergency and Organ Transplantation (D.E.T.O.), Hematology Section, University of Bari, Bari, Italy

**Keywords:** nanopore sequencing, blood diseases, target gene sequencing, structural variants, transcriptome, epigenetic modifications

## Abstract

The molecular pathogenesis of hematological diseases is often driven by genetic and epigenetic alterations. Next-generation sequencing has considerably increased our genomic knowledge of these disorders becoming ever more widespread in clinical practice. In 2012 Oxford Nanopore Technologies (ONT) released the MinION, the first long-read nanopore-based sequencer, overcoming the main limits of short-reads sequences generation. In the last years, several nanopore sequencing approaches have been performed in various “-omic” sciences; this review focuses on the challenge to introduce ONT devices in the hematological field, showing advantages, disadvantages and future perspectives of this technology in the precision medicine era.

## Introduction

The role of genetic and epigenetic alterations in hematopoietic disorders is clear but more and more detailed knowledge is still emerging. Mounting evidence in the last decade has shown that genomic data are crucial to characterize the molecular pathogenesis of hematological diseases, in multiple fields of clinical practice such as diagnosis, prognostic stratification, treatment decision, measurable residual disease detection, hematopoietic stem cell transplantation, and posttransplantation chimerism control (Koutsi and Vervesou, 2018).

The introduction of next-generation sequencing (NGS, more correctly defined as “second generation sequencing”) has considerably improved the throughput of data generated by Sanger sequencing (SS) (first-generation sequencing). NGS approaches, that are becoming ever more widespread in blood diseases research and diagnostic laboratories, have greatly increased our genomic knowledge of hematopoietic disorders (Kuo et al., 2017), but these technologies remain expensive, laborious, time-consuming, and affected by the limits of short-read sequences generation.

In this scenario, in 2012, Oxford Nanopore Technologies (ONT) released the first long-read nanopore-based sequencer, known as MinION: a handheld third generation sequencing device that works connected to a laptop (Loman and Quinlan, 2014). In 2014, ONT launched a community-focused access project: the MinION Access Programme (MAP). The nanopores sit in a membrane that separates two ionic solutions, allowing an electrical current to ﬂow through the nanopores. DNA or RNA molecules are carried through the protein pore, producing an ionic current change which is measured by a sensor with a constant sampling frequency, and later converted into nucleotides thanks to the basecalling algorithms (Goodwin et al., 2016; Magi et al., 2017). In this way, sequencing occurs without the synthesis of new strands as in the second-generation technologies.

In the last six years, the nanopore sequencing (NS) performance has been tested in various “-omic” sciences: genomics, epigenomics and transcriptomics. In this review we summarize the initial attempts to introduce the ONT platforms in the study of hematopoietic disorders, reporting the advantages and disadvantages of long-read sequencing and focusing on the future perspectives of a technology which is still emerging, but offers great promise.

### Target Gene Sequencing

NS has been widely applied for target gene sequencing (**Table 1**) and is currently the most widespread NGS application in the medical field, where the mutational status of a specific set of genes can be decisive for establishing diagnosis, prognosis, and targeted therapy.

**Table 1:** State of art and future perspectives of nanopore sequencing applications in blood diseases.

NS applications in blood diseases	State of art	Advantages	Disadvantages	Future perspectives
***Target gene sequencing***	*TP53* mutational analysis in CLL *BCR-ABL1* KD sequencing in Ph+ leukemia Gene panel sequencing (*TP53*, *SF3B1*, *MYDD88*, *BIRC3*, *NOTCH1* genes) in CLL *TP53* and *IgHV* mutational analysis in CLL *GBA* sequencing in Gaucher disease *NPM1, FLT3, CEBPA, TP53, IDH1* and *IDH2* mutational analysis in AML	Variants phasing analysis Analysis of GC-rich regions and repeated regions difficult to sequence Sequencing of complex genomic regions including internal tandem repeats or pseudogenes	Relatively high error rate, especially in homopolymers	Phasing variants on transcripts to understand the effect of the mutations on gene/isoform expression Mutational analysis of genes/genomic regions not easily to analyze with other sequencing approaches
***Structural variants characterization***	Xq28 rearrangement detection in severe HA *BCR-ABL1* genomic breakpoint characterization in CML Detection of *BCR-ABL1*, *PML-RARA*, *CBFB-MYH11*, *KMT2A-MLLT3*, *PAX5-AUTS2* and other gene fusions involved in sarcomas or lung cancers *DUSP13-GRIN2B* and *NUP98-NSD1* finding in AML del(17p13.1) and del(13q) characterization in CLL	Reads up to 2 Mb useful for SV detection More accurate identification of SV in large repetitive regions Precise identification of genomic breakpoints which are unique for each patient	Improvements required for the enrichment strategy step and in the final bioinformatics analysis	Rapid complex karyotypes characterization Detection of rearrangements not easily identifiable
***Transcriptome analysis***	Detection of *BCR-ABL1*, *PML-RARA*, *CBFB-MYH11*, *KMT2A-MLLT3*, *PAX5-AUTS2* and other gene fusions involved in sarcomas or lung cancers	Improved identification of splice junctions, specific isoforms and chimeric transcripts Estimation of the poly(A) tail length Epitranscriptome analysis Opportunity of phasing variants on the transcripts Single-cell transcriptome more efficient compared to short-read sequencing	Lower throughput compared to short-read sequencing A certain percentage of reads unlikely full-length	Unambiguous characterization and quantification of full-length isoforms and splice variants Epitranscriptomics studies
***Epigenetic modifications identification***	No applications to date	Direct sequencing of native genomic DNA to identify DNA methylations and other epigenetic marks	An efficient enrichment method not yet available	Identification of aberrant methylation patterns predicting clinical responses Study of the disease related genes transcription due to the chromatin accessibility Easy epitranscriptome analysis and its involvement in stem cell self-renewal and differentiation

NS, nanopore sequencing; CLL, chronic lymphocytic leukemia; KD, kinase domain; Ph+, Philadelphia-positive; AML, acute myeloid leukemia; HA, hemophilia A; CML, chronic myeloid leukemia.

The main NS drawback is the relatively high error rate compared to standard short-read methods (**Table 2**). Indeed, variant calling error rates from ONT data still remain relatively higher; however, starting from the first works with MinION sequencer (Ammar et al., 2015; Minervini et al., 2016), significant improvements to base-calling and single nucleotide variant (SNV)–calling steps have been made, making NS a device that may potentially be used in routine molecular tests (Minervini et al., 2016; Minervini et al., 2017; Orsini et al., 2018).

**Table 2:** Error-rate comparison of NS, PacBio, Ilumina, and Ion Torrent sequencing platforms.

	NS	PacBio	Illumina	Ion Torrent
**Read length**	Variable (200 bp up to 2 Mbps)	Up to 20 kb	Up to 600 bp (2x300 PE)	Up to 400 bp (SE)
**SNV error rate**	1%–5%	0.1%*	<0.1%	<0.1%
**Indel error rate**	5%–10%	4%*	<0.1%	1%

PE, pair-end; SE, single-end; *Error-rate estimation of PacBio circular consensus sequencing (CCS) method.

Since the first MinION release, the error rate has considerably improved thanks to changes in sequencing chemistry, the introduction of new base-calling algorithms, postsequencing correction tools and nanopore SNVs/insertions-deletions (INDELs) calling tools (Rang et al., 2018; Makałowski and Shabardina, 2019). Most of the substitution/deletion errors in nanopore reads occur in homopolymers, because their length is not correctly inferred from the electric signal (Rang et al., 2018). This issue has improved over time, reducing the SNVs/INDELs error rate from 40% to 5%–15% on average (Magi et al., 2017; Rang et al., 2018).

In general, NS and other long read sequencing platforms such as Pacific Biosciences (PacBio) suffer of a high error rate and companies are working hard to overcome this drawback. ONT improved data quality by sequencing both template and complementary strands to obtain a final more accurate consensus read; to this aim 2D and subsequently 1D^2^ library preparation protocols have been developed, although the error rate was still about 5%–10%.

Recently, PacBio has developed the Circular Consensus long-read Sequencing method (Wenger et al., 2019), able to combine the high-accuracy sequencing with long reads generation, thus reducing the mean error rate from 12%–15% to 0.1% (**Table 2**). Likewise, to improve NS results the Rolling Circle Amplification to Concatemeric Consensus has been proposed (Volden et al., 2018).

In this context, by comparing the two platforms, PacBio currently shows better data quality for the resulting consensus data; on the other hand, NS allows to obtain longer reads, provide a higher throughput, as the nanopores can sequence multiple molecules, and has more advantageous and competitive costs than PacBio sequencing.

More promising data for NS came from a new sequencing method combining unique molecular identifiers (UMI’s) with NS has been proposed (Karst et al., 2019), obtaining consensus sequences with a mean error rate of 0.03%, similar to short-read sequencing, and representing a promising strategy to improve NS data accuracy.

Furthermore, error correction tools have already been tested (Minervini et al., 2016; Orsini et al., 2018; Makałowski and Shabardina, 2019); however, further improvements would be desirable to make the NS performance comparable to standard NGS technologies.

Target enrichment is the other critical aspect of the target sequencing and is closely related to the sequencing technology. To date, the most used target enrichment approach is Polymerase Chain Reaction (PCR) based even if the capture technology is another possible alternative. Noteworthy, a PCR free target enrichment strategy based on Cas9 technology has been recently proposed (Stevensid et al., 2019). In this strategy, sample DNA is firstly 5’- dephosphorylated, to prevent the ligase binding and Cas9 is then used to cleave the DNA at predetermined sites, exposing ligatable ends. These latter are then used to attach sequencing adapters for library preparation. In this way only the region of interest is available for sequencing. Furthermore, a modified Cas9 negative enrichment strategy contemplates digestion with exonuclease after the Cas9 action. In this protocol Cas9, after the cutting step, protects the DNA target region from being digested (Stevensid et al., 2019).

#### NS in Hematology

A major advantage of NS gene mutational analysis is the possibility of phasing genetic variations, by establishing the allelic context of mutations affecting the same gene but too physically distant to be detected with the short-read methods (Minervini et al., 2017; Cumbo et al., 2019). NS also allows the analysis of GC-rich regions or repeated regions that are difficult to study with conventional NGS sequencing approaches. As regards variant calling tools, starting from the methods used in the first works, many bioinformatic resources have been developed over time (Makałowski and Shabardina, 2019); moreover, in addition to custom procedures for phasing analysis, some specific software are currently available, such as the WhatsHap tool (Martin et al., 2016).

In the blood diseases context, NS has been widely tested to evaluate the mutational status of single or multiple genes involved in a specific disease. For example, it is well known that altered p53 function, due to a 17p deletion (del(17p)) and/or *TP53* gene mutation, is associated with poor prognosis in chronic lymphocytic leukemia (CLL) patients, who are candidates for Bruton tyrosine kinase inhibitor treatment (Malcikova et al., 2018). *TP53* NS with MinION was initially performed as a single test (Minervini et al., 2016), then as part of a gene panel including the most frequently mutated genes in CLL (Orsini et al., 2018), and was finally included in a screening assay encompassing *TP53* mutational analysis, del(17p) detection, and *IgHV* mutational status evaluation (Burns et al., ). Compared to SS, *TP53* analysis by NS can be performed even from one single amplicon, instead of one PCR per exon, obtaining long reads throughout the gene; this approach is less laborious and, in cases when more than one variant is identified, allows their phasing to be established.

A nanopore assay was also developed to identify *BCR-ABL1* kinase domain (KD) mutations in Philadelphia-positive (Ph +) leukemias: chronic myeloid leukemia (CML) patients with treatment failure and acute lymphoblastic leukemia (ALL) cases at diagnosis (Minervini et al., 2017). Indeed, about 15%–30% of newly diagnosed chronic phase (CP)-CML patients will not reach an optimal response with first-line tyrosine kinase inhibitors (TKIs) therapy, and in about 25%–50% of them a *BCR-ABL1* KD mutation will be identified (Soverini et al., 2011). This frequency increases among accelerated phase (AP) and blast crisis (BC) patients (Jabbour et al., 2006). The frequency of these mutations is much higher in ALL-Ph + patients at the time of relapse than CML (Jones et al., 2008). Compared to SS, that is considered the gold standard method, *BCR-ABL1* KD NS has the advantage of potentially identifying low-level variants (< 15%–20% variant frequency, out of SS ability detection) and distinguishing between “compound mutants” (multiple mutations in the same clone) and “polyclonal mutants” (different clones) (Minervini et al., 2017). Indeed, it has been widely documented that the presence of *BCR-ABL1* KD “compound mutations” defines a subset of patients with an increased likelihood of disease progression and resistance to second generation TKIs (Parker et al., 2016; Soverini et al., 2016).

Moreover, the long-reads performances simplify some hard tasks in acute myeloid leukemia (AML) molecular evaluation.

The presence of biallelic mutations in *CEBPA* gene, identifies a subgroup of patients which is a separate entity in the recent WHO classification, with distinct biological and clinical features (Amriah, 2006; Cumbo et al., 2019). As already discussed for BCR-ABL1 short-reads methods show difficulties to phase variants and biallelic status can only be inferred. The use of a long-read technology allows a direct detection of simultaneous presence of mutations over the distance in CEBPA gene. As well, conventional NGS approaches show limitations for the detection of longer *FLT3* internal tandem duplications (ITDs) (Schranz et al., 2018) whose characterization is crucial in cytogenetically normal AML without NPM1 mutation, where *FLT3*-ITDs length was recently associated to a worse outcome (Chen et al., 2019). In the near future, targeted gene sequencing approaches may be surpassed by whole-genome sequencing (WGS) that will be used more and more frequently in clinical medicine in diagnostic contexts and to inform treatment choices (Bowden et al., 2019). Further improving base calling, alignment and variant calling methods will be crucial for this purpose. NS has also been applied to *GBA* gene sequencing (Leija-Salazar et al., 2019). Biallelic *GBA* mutations are responsible for Gaucher’s disease, the most frequent lysosomal storage disorder. *GBA* sequencing is complicated by some issues, especially the presence of a nearby pseudogene, *GBAP1*, a complex surrounding genomic region, and some exons that are difficult to sequence. In this context, long-read sequencing has proved to identify mutations that are otherwise difficult to observe, intronic variants not detectable with the conventional SS approach, and to perform phasing analysis, thus contributing to a deeper knowledge of the biological effect of *GBA* mutations.

Overall, given the low costs (from around 90€/Gb to 2€/Gb depending of the platform used) and the ease of library preparation protocols, NS could potentially enable more laboratories to perform targeted gene sequencing analysis; the introduction of user-friendly bioinformatic tools specific for NS data will further improve its diffusion in medical field.

### Structural Variants Characterization

Structural variants (SVs) of DNA are defined as regions larger than 50 bp characterized by a location shift or a change in copy number in the genome. They include translocations, inversions, insertions, deletions, duplications, expansion of repetitive sequences and combinations of these (Escaramís et al., 2015; Sudmant et al., 2015). These genomic alterations are frequently observed in hematological diseases even if their identification is sometimes difficult.

The techniques generally used in clinical practice for this purpose range from cytogenetics to molecular biology. Conventional and molecular cytogenetic techniques are rather laborious, time consuming and affected by low sensitivity; on the other hand, molecular biology approaches, although easier and more sensitive, can answer few specific diagnostic questions. The advent of NGS approaches currently used in diagnostics has not markedly improved this practice, because of the short length of reads generated. NS allows the processing of fragments of thousands of kb and the generation of long reads up to 2 Mb, a new record for a single contiguous DNA sequence (Payne et al., 2019). Clearly, this ability permits a more accurate characterization of SV especially for the assembly of large repetitive regions (De Coster and Van Broeckhoven, 2019).

Regarding the complete NS workflow for SV characterization, the two critical points to be considered are the choice of the enrichment strategy (during library preparation) and the final bioinformatic analysis. Although the targeted approach is the most used strategy to perform SV analysis, NS allows SV detection also by the WGS approach.

#### NS in Hematology

##### Targeted Approach

The superiority of single molecule long-read sequencing was demonstrated in the characterization of a new Xq28 rearrangement disrupting *F8*, in a case of genetically unresolved severe hemophilia A (HA) (Chatron et al., 2019). The study presented a 3.8‐Mb Xq11.1q12 inserted duplication in the *F8* intron 25, producing an *F8*/Xq12 (noncoding sequence) fusion transcript. Nanopore technology could improve HA molecular diagnosis, particularly in those cases (about 2%) in which conventional molecular technologies fail.

The nanopore ability to generate long-reads was also exploited to characterize the *BCR-ABL1* genomic breakpoint in ten patients affected by CML, in order to develop an approach for the “personalized monitoring” of residual disease during follow-up (Cumbo et al., 2018). NS simplifies the identification of genomic junctions that are different in each patient; this procedure is difficult and expensive to accomplish with the short-read sequencing approach and not suitable for clinical practice. The sequence thus obtained can then be used to design and test a patient-specific droplet digital PCR assay to make an absolute quantification of residual leukemic cells in patients’ peripheral blood (Cumbo et al., 2018).

The chance to detect fusion oncogenes was developed in order to develop a rapid assay for cases needing fast results (Jeck et al., 2019). The pipeline proposed was tested on a cell line (K562) and on 16 multiplexed specimens (14 clinical samples and two control analytes) for the identification of *BCR-ABL1*, *PML-RARA*, *CBFB-MYH11*, *KMT2A-MLLT3*, *PAX5-AUTS2* and other gene fusions involved in solid tumors such as sarcomas or lung cancers. Comparing with cytogenetics and molecular techniques, NS offers a more rapid approach leading to a result in two hours and sometimes in 30 minutes thanks to the possibility to analyze data in real time. Moreover, NS allows the detection of all possible fusion partnering and variants. Furthermore, authors reported the advantages of NS compared with Illumina and other NGS platforms: faster results, low initial investment costs, no need to batch samples to reduce costs and workload. The assay showed high sensitivity and specificity, with no false positives (in the high-quality calls) when compared to Illumina sequencing, even if further improvements will be required in the base-call quality step (to reduce the known higher error rates in nanopore reads) and in the development of an ecosystem of tools for real-time processing of nanopore data (to rapidly perform the relevant fusion calls).

##### WGS Approach

Recently, the rapid detection and breakpoint characterization of two chromosomal translocations in a newly diagnosed AML patient, by WGS has been reported (Au et al., 2019). A 26,194 bp sequencing read revealed the exact translocation breakpoint between *DUSP13* (chromosome 10q22.2) and *GRIN2B* (chromosome 12p13.1): a nonproductive passenger event yet identified by conventional cytogenetics; a 20,709 bp sequencing read, instead, characterizes the breakpoint between *NUP98* (chromosome 11p15.4) and *NSD1* (chromosome 5q35.3), a known cryptic driver translocation not amendable for detection by routine karyotyping. The case study suggests the potential use of NS for rapid general SV screening, even if a test with such a low depth of coverage (1x) cannot be separated from a validation step. Moreover, WGS performed in 11 CLL cases identified, with an average depth of coverage of 1.6x (range 0.1–3.4x) and an average read length of 6.3 kbp, two important chromosome deletions (Burns et al., ). In fact, in three cases del(17p13.1) were identified, 21.3 Mb, 20.3 Mb, and 22 Mb in length, respectively; instead four cases harbored del(13q), with an 800kb minimally deleted region encompassing *MIR16*, *MIR15A* and *DLEU1* (Burns et al., ). As regards the sequencing run, the accuracy of the different chemistry developed by ONT in the last years is suitable for this kind of approach. In fact, the SV detection is not affected by the relatively high error rate of the platform (De Coster et al., 2019).

### Transcriptome and Epitranscriptome Sequencing

RNA-sequencing (RNA-seq) has been a real revolution, making it possible to analyze gene expression, uncover novel RNA molecules and isoforms and identify mutations in transcripts (Pan et al., 2008; Piskol et al., 2013). Nowadays, RNA-seq methods are routinely applied for differential genes/isoforms expression analysis as well as other specific applications, such as gene fusion detection, targeted sequencing and single-cell analysis.

Up to now, short-read NGS has been used for RNA-seq; however, the main drawback of these technologies remains the prior fragmentation of the messenger RNA (mRNA) molecules, and the later computational reconstruction and assembly of the short-reads. Indeed, the assembled transcripts do not always contain the precise isoform information (for example, due to repetitive sequences or a low expression level with a consequently insufficient number of reads, or for multimapping of the short-reads) and alternative splicing patterns detection may be not accurate.

Short-read RNA-seq is also hampered by systematic issues, such as the PCR bias, and up to now all the RNA-seq preparation methods have required the conversion of RNA into complementary DNA (cDNA), amplification of these molecules and then sequencing. Each of these procedures can potentially introduce artifacts (Reid-Bayliss and Loeb, 2017).

Short-read sequencing can produce reads of up to 350 bp, but the read depth is generally high and the error rate is very low; on the other hand, long-read sequencing produces long reads with a relatively higher error rate, but good performance with splice junctions and repetitive regions resolution (Kukurba and Montgomery, 2015). Moreover, Rolling Circle Amplification to Concatemeric Consensus method (Volden et al., 2018) showed to generate more accurate reads of full-length RNA transcript isoforms than any other available long-read sequencing approaches by generating consensus sequences with increased base accuracy.

Recently, long-read sequencing has demonstrated peculiar advantages even for transcriptome analysis. In fact, this approach eliminates the need for the assembly of short-reads, ensures a more efficient alignment to repetitive regions and allows the detection of unambiguous isoforms (Stark et al., 2019).

The NS approach has been a real revolution for the RNA-seq procedure, because PCR bias can be eliminated by using direct cDNA/RNA sequencing. The main strengths of this strategy are the possibility to obtain full-length transcripts, with unambiguous identification of transcripts and isoforms, and the opportunity to study splice variants and chimeric transcripts and SVs more accurately. As in NS the read length corresponds to the fragment length, the limits typical of short-read sequencing are overcome.

Nanopore transcriptome analysis also allows estimation of the poly(A) tail length (Workman et al., 2018), that is important for RNA stability and translation, and is difficult to study with standard short-read sequencing methods (Seki et al., 2019). Overall, NS currently offers three different approaches for RNA-seq: cDNA-PCR sequencing, direct cDNA sequencing and direct RNA sequencing; the latter is the first direct RNA sequencing method (Garalde et al., 2018). Compared to short-read sequencing, long-read RNA-seq, whether long-read cDNA or direct RNA, is potentially a more appropriate approach for isoform discovery, fusion transcript discovery, *de novo* transcriptome analysis and analysis of complex loci.

Moreover, direct RNA-seq allows epitranscriptome analysis, through the detection of transcriptional modifications inferred from the signal as the RNA molecule passes through the nanopore (Schwartz and Motorin, 2017; Workman et al., 2018; Wongsurawat et al., 2018; Liu et al., 2019), as subsequently described. However, the main drawback of nanopore transcriptome analysis is a lower throughput compared to short-read sequencing; the throughput further decreases when using the direct RNA approach, that is the method with the highest input requirement, due to a slower transition through the pore compared to the DNA strand. In direct RNA-seq, 500 ng of polyA mRNA are needed as starting material, which may not be available for some critical samples. Moreover, RNA degradation can compromise an efficient detection of splice variants; the application of method for isolating intact transcripts (for instance, by targeting the eukaryotic 5′ cap) may be very effective for this purpose (Garalde et al., 2018).

In some papers, nanopore cDNA and direct RNA sequencing approaches have been compared, illustrating the strengths and limitations of both (Workman et al., 2018; Seki et al., 2019; Soneson et al., 2019). Although direct sequencing of full-length cDNA/RNA molecules is promising, it has been reported that a certain percentage of the raw nanopore reads are unlikely to be full-length reference transcripts, both with direct cDNA and RNA sequencing, thus interfering with the true identification/quantification of transcripts; this aspect needs to be improved (Soneson et al., 2019).

Regarding bioinformatics tools to be used for data analysis, dedicated pipelines/software have recently been developed and are currently available for nanopore RNA-seq analysis (Makałowski and Shabardina, 2019; Seki et al., 2019) (https://long-read-tools.org/). Some applications are more efficiently performed with NS compared to short-read sequencing: the detection of fusion transcripts, the opportunity of identifying allele-specific transcriptional events by phasing variants on the transcripts, and single-cell transcriptome analysis (Seki et al., 2019). Until now, most of the transcriptome studies in human research performed with NS namely were aimed at unambiguously characterizing and quantifying full-length isoforms and splice variants (Clark et al., 2018; Sakamoto et al., 2019; Rahimi et al., 2019; Hardwick et al., 2019), and identifying fusion transcripts and determining variants phasing (Byrne et al., 2017; Suzuki et al., 2017; Jeck et al., 2019), especially in cancer and neurological disorders.

#### NS in Hematology

In the hematological field, nanopore RNA-seq is still in its infancy, but some applications are currently emerging. By combining the MinION system and Anchored Multiplex PCR (AMP)-based libraries, a nanopore-based sequencing assay was developed to rapidly detect fusion genes in acute leukemia, where fusion detection is time sensitive and clinically relevant to choose the best treatment depending on the leukemia subtype (Jeck et al., 2019). Similarly, a NS assay based on the analysis of full-length cDNA has been developed to analyze the single-cell transcriptome, and has been applied to study the transcriptional heterogeneity of B cells with different surface receptors. Single-cell transcriptome analysis coupled with long-read sequencing makes it possible to study the real cellular transcriptional diversity at both gene and isoform level in these cells, defining and quantifying complex and/or never before reported isoforms (Byrne et al., 2017) and so extending the knowledge of physiological cellular processes and their alterations.

NS was introduced as a “validation tool” as a part of a new method, called Genotyping for Transcriptome (GoT), developed to associate the somatic genotyping of expressed genes with transcriptional profiling of single cells (Nam et al., 2019). This approach was applied to CD34+ cells from patients with myeloproliferative neoplasms (MPNs) to inspect the biological effect of calreticulin gene mutations on the cells transcriptome.

Long-read sequencing applied to transcriptome analysis may help acquire more biological information in various hematological diseases; for example, in Philadelphia chromosome (Ph)-like ALL, a crucial question is how to easily and rapidly identify Ph-like ALL (Tasian et al., 2017); gene/isoform expression analysis and fusion transcripts detection by NS may help to elucidate this aspect. In multiple myeloma (MM), where gene expression profiling has been used to identify defined molecular subgroups (Broyl et al., 2010), long-read sequencing may be useful to further improve the biological knowledge of MM cells transcriptomes.

The possibility of phasing variants on transcripts may also be exploited to understand the effect of the mutations identified in genes involved in leukemic transformation on gene/isoform expression. However, it should be underlined that the potential advantages associated with long-read analysis from blood samples have to do with the difficulty of obtaining good quality RNA from formalin-fixed paraffin-embedded (FFPE) tissue specimens from solid tumors and lymphomas.

In the future, direct RNA-sequencing may elucidate some still unknown biological issues in hematological diseases. It is known that methyladenosine (m6A) is a reversible nucleotide modification catalyzed by the METTL3/METTL14 methyltransferase complex4, that is involved in various normal biological processes, such as stem cell self-renewal and differentiation (Deng et al., 2018), as well as in AML pathogenesis (Vu et al., 2017; Barbieri et al., 2017). Several data support the concept that in AML cells the methylation of specific transcripts is crucial for AML translational regulation, and that the depletion of METTL3 can alter this process, inducing cell apoptosis in malignant cells, thereby suggesting this gene as a new potential target. In this context, direct RNA-seq could provide a direct map of m6A modifications in leukemic cells and evaluate the biological impact of this mechanism on transcriptomes in AML.

### Epigenetic Modifications Identification

DNA bases chemical modifications can influence the biological functions. Nowadays, different base modifications are described to play an important role in mammals, in physiological processes such as aging, gene regulation, imprinting, as well as disease. Methylcytosine (5-mC) is the most common among these modified bases in the genome. In addition to methylation of the cytosine (C) residue, other modifications such as oxidation of the 5-mC to 5-hydroxymethylcytosine (5-hmC), 5-formylcytosine (5-fC), 5-carboxylcytosine (5-caC), and methylation of adenine (A) to N6-methyladenine (m6A), are being identified as important epigenetic regulators (Smith and Meissner, 2013; Klungland and Robertson, 2017; Kumar et al., 2018). The cancer cell genome undergoes dramatic shifts in the pattern of genomic methylation, including genome-wide hypomethylation in conjunction with local areas of hypermethylation. Aberrant hypomethylation causes the expression of certain genes, such as oncogenes, while hypermethylation causes the inhibition of tumor suppressor genes (Feinberg and Vogelstein, 1983; Esteller et al., 2001). Additionally, the spatial and temporal regulation of DNA methylation in the hematopoietic developmental hierarchy is critical to hematopoietic homeostasis.

#### NS in Hematology

Recent studies suggest that the epigenetic factors act in concert with the transcriptional factors to ensure hematopoietic homeostasis (Cullen et al., 2014; Sashida and Iwama, 2012; Smith and Meissner, 2013; Beerman and Rossi, 2014; Gore and Weinstein, 2016; Kumar et al., 2018). An improper orchestration of the epigenetic mechanisms that control this balance can cause an aberrant hematopoietic stem cell (HSC) function and induce several hematological disorders/malignancies, such as leukemias and lymphomas, MPN, myelodysplastic syndromes (MDS), and MM. In the context of hematological malignancies, the methylation and, more generally the epigenetic status, could affect the prognosis (Toyota et al., 2001; Gore and Weinstein, 2016; Timms et al., 2016). Changes in the promoter methylation of *TAL1*, *ERHV-3*, *CDKN2A*, *CALCA*, *p15*, *IDH1/2*, and *TP53* genes have been associated with relapse in T-cell acute lymphoblastic leukemia (T-ALL) (Hogan et al., 2011; Nordlund et al., 2013). Moreover, an increasing number of recent studies are unveiling aberrant methylation patterns that predict survival in many blood malignancies, supporting the significance of measuring DNA methylation before treatment in order to predict clinical responses (Toyota et al., 2001). However, to assess the epigenomic alterations, the currently available methodologies are sophisticated and time-consuming, making them difficult to standardize and implement. Short-reads NGS strategies contemplate bisulfite conversion of unmethylated C to uracil (U) before sequencing, and so are affected by the conversion efficiency and by difficulties in repetitive genomic sequences (Barros-Silva et al., 2018). In the field of long-reads technology, a more affordable NS is also able to detect directly modified DNA and RNA bases by measuring the fluctuations in the ionic current signal between modified and unmodified bases (Simpson et al., 2017). Several bioinformatics approaches have been developed to call modified bases from the electric events obtained during the sequencing run (https://long-read-tools.org/). Basically, all such methods are able to call 5-mC but they differ as regards the training data needs and the ability to distinguish other specific modifications. The major advantage in using the nanopore approach for the base modification analysis is the possibility to directly sequence the native genomic DNA. However, when a specific target region needs to be analyzed, this advantage becomes a limit due to the lack of an efficient enrichment methodology. For this purpose, Cas9 enrichment strategy previously described, allows to overcome this issue. Apart from the methylation, other epigenetic marks could be investigated using NGS, such as the nucleosome occupancy or the histone modifications. The degree of packaging of the DNA in the chromatin has an effect on the gene expression because it controls access to the factors that regulate gene transcription. Recently, some authors have published a study on erythroid differentiation highlighting the role of the changes in the chromatin accessibility during various stages of erythropoiesis (Ludwig et al., 2019). Furthermore, other authors showed specific increases of chromatin accessibility in MM as compared to normal B cells. These changes cause enhancer activation and disease-related genes transcription (Jin et al., 2018). For this purpose, some authors applied nanopore detection of the 5-mC, to define the chromatin accessibility using a method already known as NOME-seq (Kelly et al., 2012; Lee et al., 2019). NOME-seq uses GpC methyltransferase (M.CviPI) and NGS to generate a high resolution footprint of the nucleosome position. The technique allows simultaneous detection of the nucleosome occupancy and the DNA methylation pattern. Using long-read NS, the nucleosome occupancy can be observed on the single read. Furthermore, the authors propose the use of different methyltransferases, such as EcoGII which methylates A to m6A in order to obtain a “multicolor” measurement which could allow discrimination between endogenous 5-mC and induced modifications (Lee et al., 2019).

### Closing Remarks

The advent of third generation sequencing can be considered a revolution in NGS technologies. What was considered impossible only a few years ago in terms of throughput, potential, ease of use and costs, today is becoming achievable. Genetic and genomic approaches are now a routine part of biological research and are becoming ever more widespread in clinical practice. As regards hematological diseases, and especially in the context of blood malignancies, several NS approaches have been performed with the aim of elucidating some still unknown biological issues and testing the performance of the ONT platforms in diagnostic contexts (**Table 1**). As previously discussed, many advantages are to be gained from long-read sequencing: efficient variants-phasing, the ability to analyze GC-rich or repeated regions, easy characterization of the genomic SVs and the identification of full-length transcripts and isoforms. Furthermore, the ONT peculiarity of being able to directly sequence the nucleic acids allows a better study of splice variants and chimeric transcripts, and direct detection of the epigenetic modifications of DNA and RNA molecules. The scalability of the technology makes it possible to perform different approaches in terms of throughput (2 Gb–10.5 Tb), ranging from rapid target gene sequencing to WGS or whole transcriptome analysis. ONT released the GridION and PromethION platforms, allowing higher throughput sequencing runs (from 250 Gb to 10.5 Tb, respectively, according to Oxford Nanopore information); on the other hand, MinION can be adapted for smaller rapid experiments using Flongle: a single use flow cell that is ideal for target sequencing (throughput: 2 Gb) (Kumar et al., 2019). These emerging strengths, together with the constant improvements in nanopore technology may, in the near future, pave the way for the introduction of ONT in hematological research and diagnostic laboratories. In the precision medicine era, a platform with this kind of performance will help to increase the understanding of the “-omic” mechanisms in these diseases, providing clinicians with valid support in diagnosis and prognosis, thus promoting the personalized treatment of patients.

## Author Contributions

CM, CC, and PO conceived and wrote the review. LA and AZ performed the literature analysis. FA and GS supervised and approved the final manuscript.

## Conflict of Interest

The authors declare that the research was conducted in the absence of any commercial or financial relationships that could be construed as a potential conflict of interest.
